# Pregnant women at risk for iodine deficiency but adequate iodine intake in school-aged children of Zhejiang Province, China

**DOI:** 10.1007/s10653-024-01934-3

**Published:** 2024-05-02

**Authors:** Canjie Zheng, Zhiying Yin, Bingdong Zhan, Wenjie Xu, Zheng Feei Ma

**Affiliations:** 1 Quzhou Center for Disease Control and Prevention, 154 Xi’an Road, Quzhou, 324000 Zhejiang Province China; 2 Centre for Public Health and Wellbeing, School of Health and Social Wellbeing, College of Health, Science and Society, University of the West of England, Bristol, UK

**Keywords:** Iodine deficiency, Median urinary iodine concentration, Salt iodine concentration, Pregnant women, School-aged children

## Abstract

The median urinary iodine concentration (UIC) of school-aged children has been commonly used as a surrogate to assess iodine status of a population including pregnant women. However, pregnant women have higher iodine requirements than children due to increased production of thyroid hormones. The aim of the study was to evaluate the iodine status of pregnant women and children as well as their household salt iodine concentration (SIC) in Quzhou, Zhejiang Province, China. Eligible pregnant women and children from all six counties of Quzhou in 2021 were recruited into the study. They were asked to complete a socio-demographic questionnaire and provide both a spot urine and a household table salt sample for the determination of UIC and SIC. A total of 629 pregnant women (mean age and gestation weeks of 29.6 years and 21.6 weeks, respectively) and 1273 school-aged children (mean age of 9 years and 49.8% of them were females) were included in the study. The overall median UIC of pregnant women and children in our sample was 127 (82, 193) μg/L and 222 (147, 327) μg/L, respectively, indicating sufficient iodine status in children but a risk of mild-to-moderate iodine deficiency in pregnant women. Distribution of iodine nutrition in children varied significantly according to their sex and age (*P* < 0.05). The rate of adequately household iodised salt samples (18–33 mg/kg) provided by pregnant women and children was 92.4% and 90.6%, respectively. In conclusion, our results indicated a risk of insufficient iodine status in pregnant population of China, but iodine sufficiency in school-aged children. Our data also suggested that median UIC of children may not be used as a surrogate to assess iodine status in pregnant women.

## Introduction

Iodine is needed by the thyroid gland for thyroid hormone synthesis. Inadequate iodine intake can result in a decrease of thyroid hormone production, and cause significant adverse health effects (i.e., iodine deficiency disorders (IDD)), especially in pregnant women and children (Hatch-McChesney & Lieberman, 2022). Iodine deficiency has been reported to affect about one-third of the world’s populations (Ma & Skeaff, 2017). Iodine deficiency during pregnancy has been reported to be associated with increased risks of congenital hypothyroidism, neonatal hypothyroidism and neuropsychomotor retardation (Toloza et al., 2020). In a meta-analysis with 37 studies of 12,291 Chinese children, children exposed to iodine deficiency (with a median urinary iodine concentration (UIC) of < 100 µg/L) were demonstrated to have 12.5 IQ points lower than children living in iodine sufficient regions (with a median UIC of ≥ 100 µg/L) (Qian et al., 2005).

One of the most commonly used and recommended biochemical methods by the WHO/UNICEF/ICCIDD to assess population iodine status is UIC, which is determined from the spot urine samples (Ma & Skeaff, 2017; Watts et al., 2020). The collection of urine samples is preferable in the field than other biochemical methods that involve the collection of blood samples such as thyroid-stimulating hormone (TSH) (except in neonates) and thyroid hormones when assessing population iodine status. This is because it is non-invasive and easier for individuals to provide (Huang et al., 2018). In addition, UIC reflects the recent iodine status in populations because most of the ingested iodine (> 90%) is excreted in the urine of iodine replete individuals over 24–48 h (Ma & Skeaff, 2017). However, UIC is associated with high intra- and inter-individual variations (Ma et al., 2018).

In the 1980s, IDDs were identified as a severe public health in China because it affected approximately 425 million individuals and 15% of children were diagnosed with mild mental impairment (Endemic Disease Control Department, 1989; Lou et al., 2020; Sun et al., 2017). For example, Zhejiang, an eastern coastal province in China with low soil and water iodine concentrations, had approximately 12 million individuals who were at risk of IDD and 32.6% of children were diagnosed for goitre before the implementation of mandatory universal salt iodisation (USI) policy in 1995 (Huang et al., 2006). In 2010, the elimination of IDD was reported to be achieved in all provinces of China (Sun et al., 2017). However, following the increased iodine intake resulting from the USI policy, some regions in China were reported to have excessive iodine intake, which has raised another public health concern (Liu et al., 2020). Therefore, in 2012, the SIC of table salt in Zhejiang province was decreased from 35 mg/kg (range: 20–50 mg/kg) to 25 mg/kg (range: 18–33 mg/kg) to prevent individuals from consuming too much iodine through table salt (Yu et al., 2020).

Although Zhejiang province has now been categorised as iodine sufficient and met the goal of eliminating IDD since 2011 based on the median UIC of school-aged children, it remains unclear if the median UIC of school-aged children could be used as a surrogate to monitor iodine status in pregnant women in Zhejiang (Sun et al., 2017). In addition, some previous studies in China have reported the emergence of mild iodine deficiency in pregnant women where the regions are considered iodine sufficient based on the median UIC of children (Wang et al., 2017, 2018a, 2018b, 2019; Yu et al., 2020; Zhou et al., 2019, 2020). One of the possible reasons is that pregnant women have higher iodine requirements (i.e., the recommended iodine intake of 250 μg/day) to ensure the increased production of thyroid hormones and renal iodine clearance (Andersson et al., 2007). As a result, they are vulnerable to iodine deficiency (Brough, 2021).

The aim of this study was to assess iodine status of pregnant women and school-aged children as well as their household salt iodine concentration in Zhejiang province, China as part of our ongoing IDD monitoring programme.

## Materials and methods

**Fig. 1:**
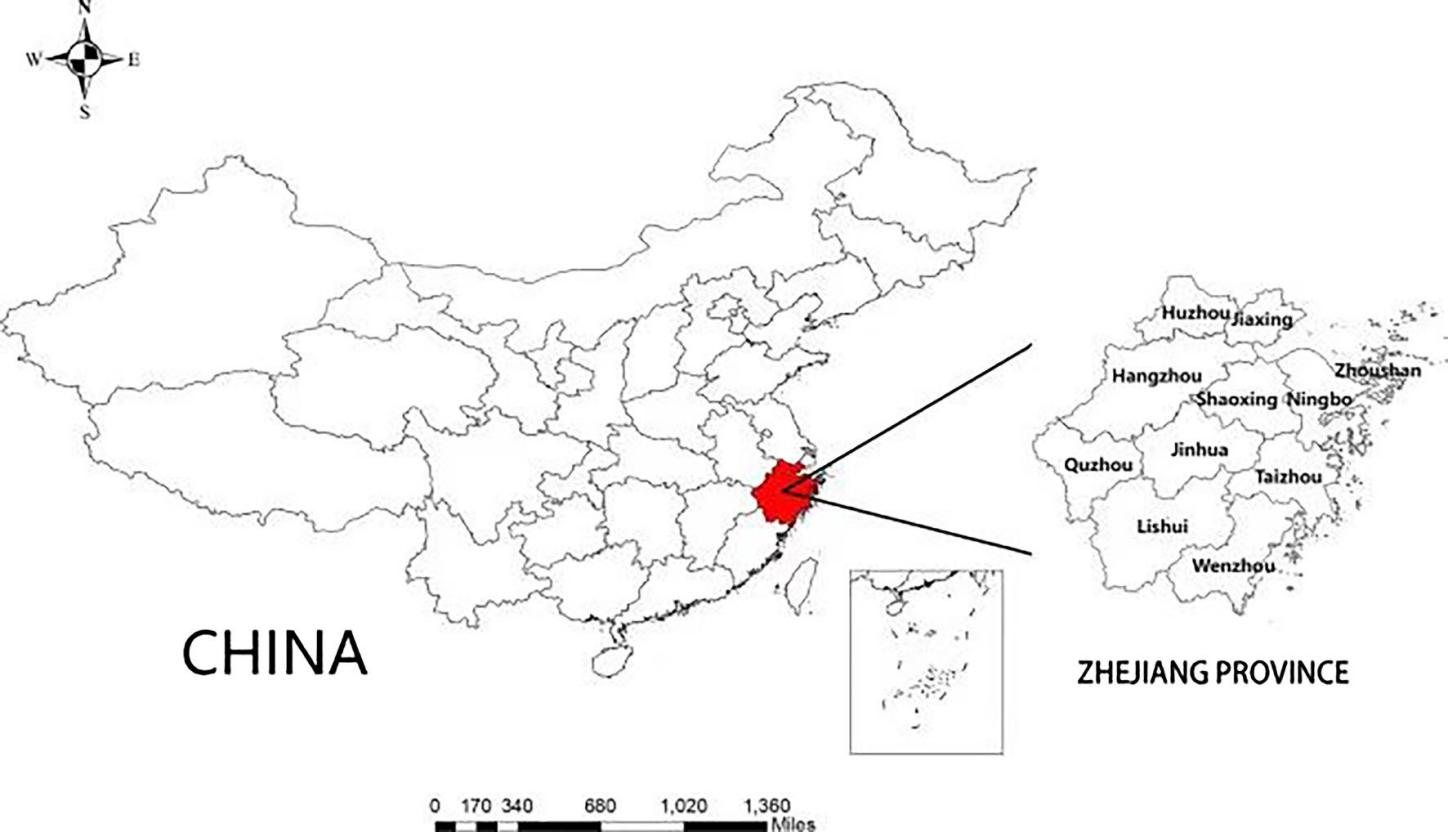
The sample locations in Quzhou, Zhejiang Province, and China

The cross-sectional study was carried out in Quzhou (with ~ 2.5 million people), Zhejiang Province, China in 2021 (Fig. 1). Local pregnant women and school-aged children were recruited from all six counties (cities and districts) of Quzhou, with each county selecting one township from the directions of east, south, northwest, and central, resulting in a total of 30 townships being chosen using a stratified probability-proportionate-to-size (PPS) cluster design based on the estimated prevalence of iodine deficiency in these population groups (Yu et al., 2020). Pregnant women were invited to participate in the study if they aged ≥ 18 years and lived in Quzhou for more than 1 year. Children were eligible for the study if they were aged between 8 and 10 years, lived in Quzhou for more than 1 year and had parental consent. Pregnant women and children were excluded if they (pregnant women and the parents of children) were unable to provide informed consent. The study had been approved by the Quzhou Centre for Disease Control and Prevention (reference no. 2021/5/4) and performed according to the Declaration of Helsinki. Informed consent was obtained from all pregnant women and the parents of school-aged children prior to the study enrolment.

### Socio-demographic data collection

Pregnant women and children were interviewed by the investigators to obtain their sociodemographic data. Pregnant women were asked to provide their age, current residency, gestation weeks and history of thyroid disease. In addition, they were asked if they used iodised salt at home.

### Assessment of iodine status

Pregnant women and children were asked to collect a 10 mL non-fasting spot urine sample using a urine container with screw cap. The urine containers were manufactured to be sterile, tested for iodine contamination and intended for single use only. All urine samples were stored at − 20 °C until analysis using the spectrophotometry method with ammonium persulphate digestion (WS/T 107.1–2016) in Quzhou Center for Disease Control and Prevention (Yu et al., 2020).

Since iodine status cannot be assessed in individuals but only as groups, the UIC cut-offs used to assess iodine status are population-based. The UIC cut-offs based the WHO/UNICEF/ICCIDD criteria for determining iodine status during pregnancy in a group of pregnant women are as follows: severe-to-moderate iodine deficiency (median UIC of < 100 μg/L), mild iodine deficiency (median UIC of 100–149 μg/L), sufficient iodine status (median UIC of 150–249 μg/L), more than adequate iodine status (median UIC of 250–499 μg/L) and excessive iodine status (median UIC of ≥ 500 μg/L) categories (Andersson et al., 2007; Xiao et al., 2018). On the other hand, the median UIC cut-offs for assessing iodine status in a group of school-aged children are as follows: severe iodine deficiency (median UIC of < 20 μg/L), moderate iodine deficiency (median UIC of 20–49 μg/L), mild iodine deficiency (median UIC of 50–99 μg/L), sufficient iodine status (median UIC of 100–299 μg/L), and excessive iodine status (median UIC of ≥ 300 μg/L (WHO/UNICEF/IGD 2007; Zimmermann et al., 2013). For the purpose of conducting a detailed analysis of UIC in this study, the single category of median UIC in children within the range of 100–299 µg/L (indicative of sufficient iodine status), as suggested by Zimmermann et al., is divided into two categories: adequate iodine status (median UIC of 100–199 μg/L) and more than adequate (median UIC of 200–299 μg/L) (WHO/UNICEF/IGD 2007;; Zimmermann et al., 2013).

Quality control samples containing known concentrations of iodine were included in the same run for the measurement of UIC, with a recovery of 97–110%. The assay internal validity was assessed using internal pooled urine sample included in each of batch of the urine sample analysis (n = 20). Analysis of the internal pooled urine sample provided a mean ± standard deviation (SD) UIC of 138 ± 4 μg/L (CV = 4.5%; n = 51).

### Assessment of salt iodine concentration (SIC)

All women and children were asked to provide a household table salt sample. The household table salt iodine concentration (SIC) was measured by an electronic titrator according to the iodometric titration method with thiosulphate (GB/T 13,025.7–2012) (Yu et al., 2020). The SIC were classified into four different groups: non-iodised (< 5 mg/kg), inadequately iodised (5–17.9 mg/kg), adequately iodised (18–33 mg/kg), and excessively iodised (> 33 mg/kg) (Mao et al., 2018; Yu et al., 2020).

All laboratory analyses and calibration of the equipment were performed according to the manufacturers’ procedures.

### Statistical analysis

Statistical analysis was conducted using IBM SPSS version 27 (IBM Corp., Armonk, NY). Values in text are presented as mean ± SD for normally distributed variables or median (25th, 75th percentiles) (IQR) for non-normally distributed variables. Chi-square tests were employed to compare the proportions. General linear model (GLM) multivariate analysis was used to determine the differences in quantitative variables. Correlations between variables were assessed using Spearman correlations. A *P*-value < 0.05 was considered statistically significant.

## Results

A total of 629 pregnant women and 1273 school-aged children were included in the final sample size (Table 1). The mean age of pregnant women and children was 29.6 ± 4.9 and 9.0 ± 0.8 years, respectively. The mean gestation week for the pregnant women was 21.6 ± 9.6 weeks. There were 36.2% of pregnant women in second trimester, followed by third trimester (32.3) and first trimester (31.5%). More than half of the children were males (50.2%). In terms of age distribution among the children, the three age groups (8, 9, and 10 years old) show similarity in their percentage representation (33.4%, 33.3%, and 33.3%, respectively).

**Table 1:** Characteristics of pregnant women and children in the study^a^

	Values
Pregnant women	
*n*	629
Age (yrs)	29.6 ± 4.9
Gestational age (wks)	21.6 ± 9.6
Trimester of pregnancy, n (%)	
1st	198 (31.5)
2nd	228 (36.2)
3rd	203 (32.3)
Children	
*n*	1273
Sex, *n* (%)	
Female	634 (49.8)
Male	639 (50.2)
Age (yrs)	9.0 ± 0.8
Age distribution (yrs), n (%)	
8	425 (33.4)
9	424 (33.3)
10	424 (33.3)

*n*, number; wks, weeks; yrs, years

^a^Mean ± SD (all such values)

The overall median UIC (IQR) of pregnant women was 127 (82, 193) μg/L, which was indicative of mild iodine deficiency (median UIC of 100–149 μg/L) (Andersson et al., 2007) (Table 2). On the other hand, the overall median UIC of children was 222 (147, 327) μg/L, indicating more than adequate iodine status (i.e., median UIC of 200–299 ug/L) (WHO/UNICEF/IGD 2007) (Table 3).

**Table 2:** Categorisation of iodine status of pregnant women by trimester of pregnancy^a,b^

	Trimesters (n = 629)			*P*-value
	1st (n = 198)	2nd (n = 228)	3rd (n = 203)	
Median UIC (µg/L)	136 (97, 200)	120 (76, 196)	124 (75, 189)	0.145
UIC (µg/L), *n* (%)				
< 50	11 (5.6)	16 (7.0)	17 (8.4)	0.551
50–99	44 (22.2)	67 (29.4)	62 (30.5)	
100–149	60 (30.8)	57 (25.0)	52 (25.6)	
150–249	61 (30.8)	62 (27.2)	45 (22.2)	
250–499	19 (9.6)	23 (10.1)	24 (11.8)	
≥ 500	3 (1.5)	3 (1.3)	3 (1.5)	
SIC (ppm)	23.2 ± 6.1	23.6 ± 5.7	23.1 ± 6.3	0.605
SIC (mg/kg), *n* (%)				
18	13 (6.6)	13 (5.7)	15 (7.4)	0.750
18–33	182 (91.9)	214 (93.9)	185 (91.1)	
> 33	3 (1.5)	1 (0.4)	3 (1.5)	

UIC, urinary iodine concentration; SIC, salt iodine concentration

^a^Median (25th, 75th percentiles) (all such values)

^b^Mean ± SD (all such values)

**Table 3:** Categorisation of iodine status of children by sex^a,b^

	Males (n = 639)	Females (n = 634)	*P*-value
Median UIC (µg/L)	228 (152, 345)^1^	218 (136, 306)	**0.020**
UIC (µg/L), *n* (%)			
< 20	1 (0.2)	11 (1.7)	**0.002**
20–49	11 (1.7)	26 (4.1)	
50–99	53 (8.3)	62 (9.8)	
100–199	200 (31.3)	182 (28.7)	
200–299	173 (27.1)	184 (29.0)	
≥ 300	201 (31.5)	169 (26.7)	
SIC (ppm)	22.2 ± 6.2^2^	22.9 ± 5.6	**0.030**
SIC (mg/kg), *n* (%)			
< 18	67 (10.5)	47 (7.4)	0.095
18–33	568 (88.9)	585 (92.3)	
> 33	4 (0.6)	2 (0.3)	

Bold values indicate that a *P*-value < 0.05 is statistically significant

UIC, urinary iodine concentration; SIC, salt iodine concentration

^a^Median (25th, 75th percentiles) (all such values)

^b^Mean ± SD (all such values)

The coverage of iodised salt was 100% in both pregnant women and children. The overall mean household SIC for pregnant women and children was 23.3 ± 6.0 mg/kg and 22.6 ± 5.9 mg/kg, respectively, suggesting that the household salt samples used were adequately iodised. There were 92.4% of pregnant women and 90.6% of children who used adequately iodised salt in their households. The mean SIC of the household salt samples provided by female children was significantly higher than that of male children (22.9 ± 5.6 mg/kg vs. 22.2 ± 6.2 mg/kg) (*P* = 0.030). The household SIC was not associated with the median UIC of children and pregnant women (all *P* > 0.05).

### Iodine status of pregnant women by trimester of pregnancy

The median UIC of pregnant women in the first trimester was 136 μg/L, which was slightly higher than those in second and third trimesters (120 and 124 μg/L, respectively) (Table 2). However, the median UIC of pregnant women did not differ among the trimesters (*P* = 0.145).

**Fig. 2:**
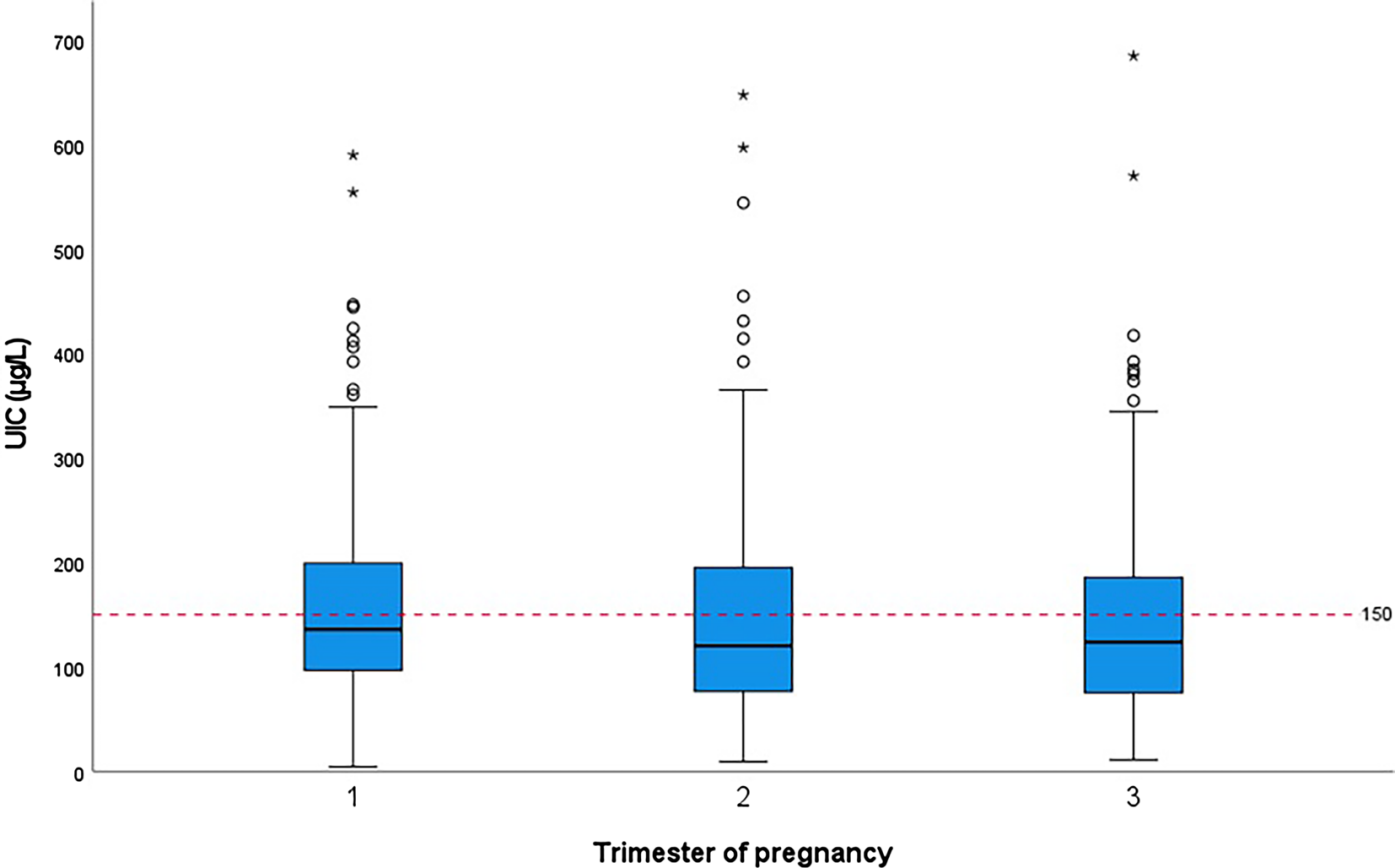
Distribution of UIC among different trimesters of pregnancy. The red horizontal dashed line indicates the median UIC cut-off value for pregnant women

Figure 2 shows the UIC distribution among different trimesters of pregnancy. There was no difference in the UIC distribution of pregnant women among the trimesters (*P* = 0.551). No association was reported between median UIC of pregnant women and gestational week.

### Iodine status of children

The median UIC of male children was higher than female children (228 vs. 218 μg/L) (*P* = 0.020) (Table 3). Female children were found to have a higher risk of inadequate iodine intake than male children (*P* = 0.002).

**Fig. 3:**
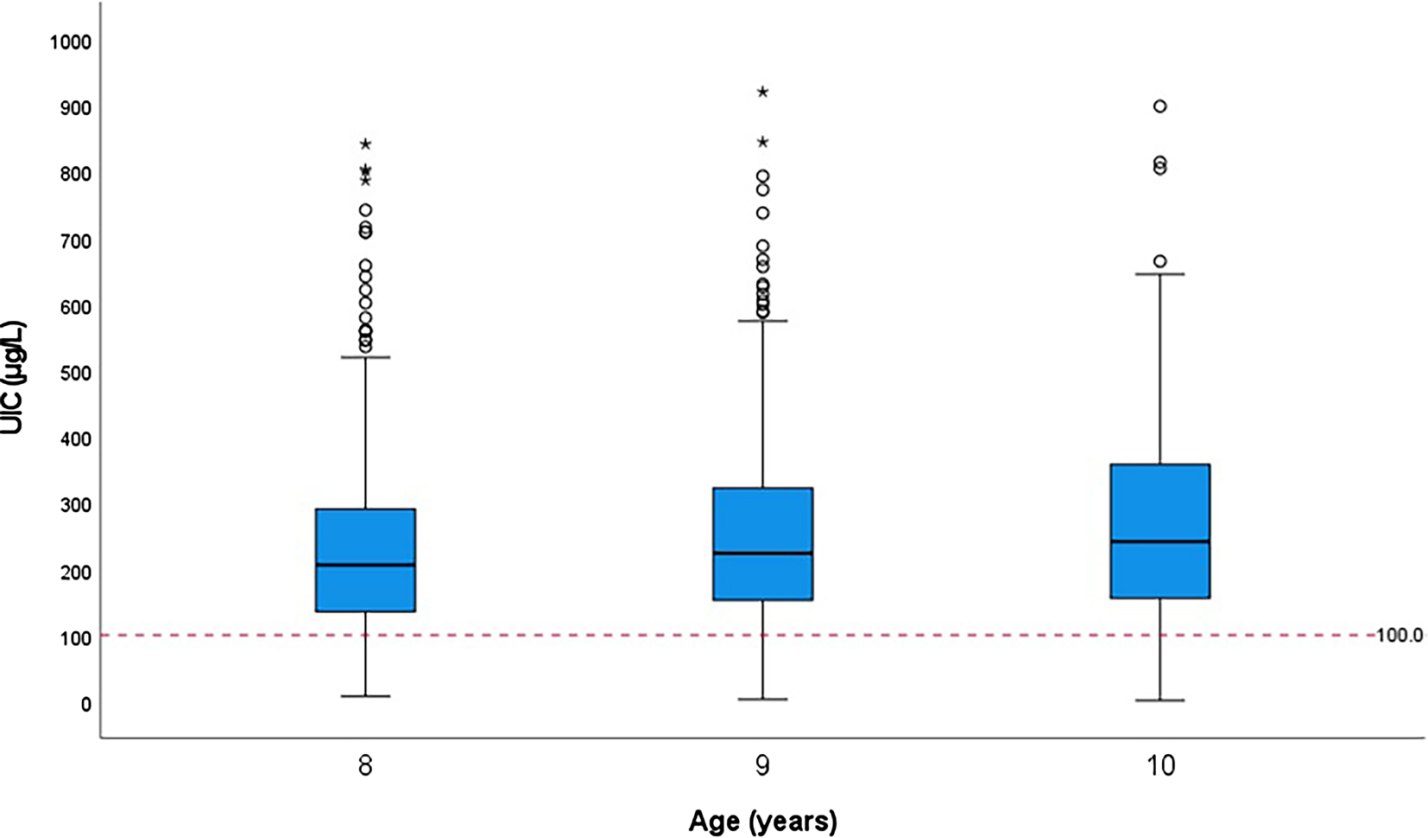
Distribution of UIC for children. The red horizontal dashed line indicates the median UIC cut-off value for children

Figure 3 shows the UIC distribution among school-aged children. Children aged 10 years had a higher median UIC than those aged 8 years (241 vs. 206 μg/L) (*P* = 0.001), but there was no difference in median UIC for children aged 9 years (224 μg/L) with those aged 8 years and 10 years (*P* = 0.235 and 0.206, respectively) (Table 4). Children aged 8 and 9 years were more likely to have sufficient iodine status than those aged 10 years (*P* = 0.001). There was no association for the categorisation of the household SIC with sex and age group of children (all *P* > 0.05).

**Table 4:** Categorisation of iodine status of children by age distribution^a,b^

	Age distribution for children (n = 1273)			*P*-value
	8 (n = 425)	9 (n = 424)	10 (n = 424)	
Median UIC (µg/L)	206 (135, 291)	224 (153, 322)	241 (156, 358)	**0.002**
UIC (µg/L), *n* (%)				
< 20	7 (1.6)	2 (0.5)	3 (0.7)	**0.001**
20–49	14 (3.3)	12 (2.8)	11 (2.6)	
50–99	41 (9.6)	27 (6.4)	47 (11.1)	
100–199	141 (33.2)	141 (33.3)	100 (23.6)	
200–299	127 (29.9)	125 (29.5)	105 (24.8)	
≥ 300	95 (22.4)	117 (27.6)	158 (37.3)	
SIC (ppm)	22.8 ± 6.1	22.7 ± 5.3	22.2 ± 6.2	0.265
SIC (mg/kg), *n* (%)				
< 18	34 (8.0)	36 (8.5)	44 (10.4)	0.128
18–33	388 (91.3)	385 (90.8)	380 (89.6)	
> 33	3 (0.7)	3 (0.7)	0 (0.0)	

Bold values indicate that a *P*-value < 0.05 is statistically significant

UIC, urinary iodine concentration; SIC, salt iodine concentration

^a^Median (25th, 75th percentiles) (all such values)

^b^Mean ± SD (all such values)

## Discussion

Iodine status of school-aged children has been recommended to represent iodine status of a population because of the easier access to this population group and they are presumed to be eating the same household foods as adults (WHO/UNICEF/IGD 2007). Therefore, the median UIC of school-aged children has been used to represent the iodine status of all population groups including pregnant women in majority of the national iodine deficiency disorders (IDD) surveys (Jiang et al., 2019; Ma & Skeaff, 2017). One of the possible reasons why this assumption is used might be that the studies on iodine status on pregnant women both at national and international levels are limited in the past (Pearce et al., 2016). It is also assumed that in regions where the implementation of universal salt iodisation has been more than 2 years and median UIC of children is considered iodine sufficient, all population group in the regions would meet the iodine requirement (Dold et al., 2018). As a result, the median UIC of children from the same region may have been used to reflect iodine status of pregnant women (Pearce et al., 2016). However, there have been increasing studies that suggested that this assumption may not be valid for pregnant women (Ategbo et al., 2008; Gowachirapant et al., 2009; Hess et al., 2017; Kuay et al., 2021; Zimmermann et al., 2005).

In our study, the median UIC in our school-aged children was 222 μg/L, indicating a risk of more-than-adequate iodine intake (WHO/UNICEF/IGD 2007). However, the UIC categories of ‘adequate iodine status’ and ‘more than adequate’ have been suggested to be merged for forming a new single category of sufficient iodine status (median UIC of 100–299 μg/L). This is because the ‘more than adequate’ category is not associated with adverse effects on thyroid function in children (Zimmermann et al., 2013). Therefore, based on the median UIC of our school-aged children, our region would be classified as iodine sufficient (Zimmermann et al., 2013). In sharp contrast, the median UIC of 127 μg/L in our pregnant women in the same region indicated a risk of mild iodine deficiency (WHO/UNICEF/IGD 2007). Our findings suggested that the median UIC of children may not be used to accurately reflect the iodine status of pregnant women from the same region. One possible reason may be due to the differences in urine volumes. Under the similar iodine intake consumption, children with smaller daily urine volumes would be more likely to have higher median UIC than pregnant women with higher urine volumes (Beckford et al., 2020; Gowachirapant et al., 2009). Similar findings have also been reported in other regions of China including Zhejiang and Shanghai (Lou et al., 2020; Wang et al., 2018a). Our results were also consistent with other findings reported in other countries including India, Malaysia, Niger, Switzerland, Thailand (Ategbo et al., 2008; Gowachirapant et al., 2009; Hess et al., 2017; Kuay et al., 2021; Zimmermann et al., 2005). On the other hand, it is possible that these children did not have excessive iodine intake. The single UIC value was high which could be due to the food they ate the day before the urine sample collection.

Our study reported the pregnant women were at risk of iodine deficiency. There have been several possible reasons that might contributed to these findings. Following the introduction of new provincial standard for the household iodised salt (25 mg/kg, with a range of 18–33 mg/kg), iodine deficiency has been reported in pregnant women in some provinces including Zhejiang, Jiangsu and Fujian (Lin et al., 2021; Wang et al., 2018b; Yu et al., 2020; Zhou et al., 2019, 2020). Other factors such as increasing availability of non-iodised salt in the market and the perception that iodine intake from fish and seafood products is sufficient to meet the iodine requirements for individuals living in coastal cities may have contributed to lower iodine intake in pregnant women (Zhang et al., 2023). In addition, women might have reduced the use of iodised salt during pregnancy because of the perception that the use of iodised salt is attributed to the rising incidence of thyroid diseases (Shaikh et al., 2022).

Our study also found that about one-third of children had a risk of excessive iodine status (≥ 300 μg/L). One of the possible reasons may be due to the high consumption of salt by in the Chinese population (~ 11 g/day) (Tan et al., 2022). Therefore, children may also consume high levels of salt since their meals are typically prepared by their parents who live in the same households (Mahmood et al., 2021). The issue of excessive iodine in children should be noted, as it can lead to the development of iodine-induced hypothyroidism due to a failure to escape the Wolff-Chaikoff effects (Lee et al., 2023).

Strengths of our study included the use of a large, population-based sample in the region. Iodine status assessment of pregnant women and children by spot urine samples was coupled with the assessment of household SIC. In addition, household salt samples were obtained from the households of pregnant women and children, which allowed further analysis of the relationships for the use of iodised salt with iodine status of pregnant women and children. One limitation of our study was that no measurement of thyroid function and thyroglobulin (Tg) was performed on pregnant women and children because Tg has been reported to a more sensitive biomarker of iodine status (Ma & Skeaff, 2014, 2017). Tg concentrations increases in iodine deficient individuals than those who are iodine sufficient (Ma & Skeaff, 2014; Ma et al., 2016). Dietary assessment was not performed on pregnant women and children in this study. Since children spent most of their time in school, future studies should consider investigating the effect of iodine in school meal on the iodine status of school-aged children. Although no urinary creatinine measurement was included, we acknowledged that hydration status may have a large impact on the perceived iodine status in a population. Future studies should consider measuring biomarkers of hydration status such as 24-h urine volume for allowing a more accurate assessment of iodine status in these populations.

## Conclusions

Our findings indicated that the median UIC in school-aged children may not be a sensitive surrogate to monitor and evaluate iodine status in pregnant women. This is because the median UIC of children may underestimate the prevalence of iodine deficiency in pregnant women as demonstrated in our study. Therefore, the assessment of iodine status in populations should not only based on the median UIC of children, but also include the monitoring of iodine status in pregnant women. Our findings need to be confirmed in other populations with varying dietary iodine intake. In addition, our results also underline the need for an ongoing monitoring of iodine status in pregnant women and children from different regions within Zhejiang Province.
